# Light Perception in Two Strictly Subterranean Rodents: Life in the Dark or Blue?

**DOI:** 10.1371/journal.pone.0011810

**Published:** 2010-07-28

**Authors:** Ondřej Kott, Radim Šumbera, Pavel Němec

**Affiliations:** 1 Department of Zoology, Faculty of Science, University of South Bohemia, České Budějovice, Czech Republic; 2 Department of Zoology, Faculty of Science, Charles University in Prague, Prague, Czech Republic; University of Lethbridge, Canada

## Abstract

**Background:**

The African mole-rats (Bathyergidae, Rodentia) are strictly subterranean, congenitally microphthalmic rodents that are hardly ever exposed to environmental light. Because of the lack of an overt behavioural reaction to light, they have long been considered to be blind. However, recent anatomical studies have suggested retention of basic visual capabilities. In this study, we employed behavioural tests to find out if two mole-rat species are able to discriminate between light and dark, if they are able to discriminate colours and, finally, if the presence of light in burrows provokes plugging behaviour, which is assumed to have a primarily anti-predatory function.

**Methodology/Principal Finding:**

We used a binary choice test to show that the silvery mole-rat *Heliophobius argenteocinereus* and the giant mole-rat *Fukomys mechowii* exhibit a clear photoavoidance response to full-spectrum (“white”), blue and green-yellow light, but no significant reaction to ultraviolet or red light during nest building. The mole-rats thus retain dark/light discrimination capabilities and a capacity to perceive short to medium-wavelength light in the photopic range of intensities. These findings further suggest that the mole-rat S opsin has its absorption maximum in the violet/blue part of the spectrum. The assay did not yield conclusive evidence regarding colour discrimination. To test the putative role of vision in bathyergid anti-predatory behaviour, we examined the reaction of mole-rats to the incidence of light in an artificial burrow system. The presence of light in the burrow effectively induced plugging of the illuminated tunnel.

**Conclusion/Significance:**

Our findings suggest that the photopic vision is conserved and that low acuity residual vision plays an important role in predator avoidance and tunnel maintenance in the African mole-rats.

## Introduction

Sensory perception plays a crucial role in animal spatial and temporal orientation, foraging and communication with conspecifics. Animals have to find and recognize a mate, kin, intruders or danger. Among senses, vision is important for many species and it has probably been a key selective advantage throughout the animal evolution [1], [2]. In the context of visual ecology, the subterranean niche is arguably the most extreme sensory environment, being deprived of light and all visual cues available above ground. However, because it provides shelter from predation and climatic fluctuations, about 250 mammalian species have adopted subterranean lifestyle [3]–[5]. The darkness of the underground ecotope not only relaxes the selection acting on the visual system, but through the metabolic gain yielded by visual system reduction also imposes a selective pressure acting in favor of its regression [3], [6]. Indeed, strictly subterranean mammals are congenitally microphthalmic and possess a severely reduced visual system, although the degree of reduction vary substantially among species (for review, see [7], [8]).

The African mole-rats (Bathyergidae, Rodentia) are strictly subterranean rodents endemic to sub-Saharan Africa. They inhabit extensive burrow systems isolated from the aboveground environment by mounds of soil and feed almost exclusively on geophytes, i.e., plants with subterranean storage organs [9]. In most species, the above ground activities appear to be restricted to rare events such as dispersal and mate-seeking excursions. Despite their strictly subterranean mode of life, the African mole-rats possess small, superficially located and structurally normal eyes [8], [10]–[12]. Nevertheless, the image-forming vision is compromised due to the properties of the optical apparatus [12], very low visual acuity [8], and, at least in some species, also due to disorganized lens [13] and retinal architecture [14]. Bathyergids have rod-dominated retinae but possess significant cone populations (∼10% of photoreceptors are cones) [10], [11]. Nearly all cones express a short-wave-sensitive (S) opsin (commonly blue- or ultraviolet-sensitive in mammals). Many of these S cones co-express small amounts of a middle-to-long-wave-sensitive (L) opsin (commonly green- or yellow-sensitive in mammals), but there are only few pure L cones [11]. Rhodopsin has its absorption peak (λ_max_ inferred from amino acid sequence) at 496–498 nm [15], the exact spectral tuning of the cone opsins is not known. Paradoxically, the eye seems to be adapted to bright-light rather than low-light conditions. Bathyergids feature small lenses that collect light rather ineffectively (e.g., [8]), high cone proportions [11] and rod nuclei with conventional architecture (Němec et al., unpublished data; cf., [16]). All these features are characteristic of diurnal mammals and are not expected in animals adapted to subterranean darkness.

The central visual system of bathyergids has undergone mosaic regression [17]–[19]. The only well developed visual domains are those involved in controlling the circadian and circannual biological rhythms – the suprachiasmatic nucleus and the retinohypothalamic projections. The lateral geniculate body (a relay nucleus for cortical perception subserving the detection of colour, form and motion) and pretectum (involved in luminance detection and the pupillary light reflex) are only moderately reduced. By contrast, the superficial visual layers of the superior colliculus (which exerts an important function in object localization) and the accessory optic system (used to stabilize the image on the retina during head movements) are vestigial. This indicates that the bathyergid mole-rats are poorly equipped for the detection and orientation towards objects in the visual field, and for the tracking of moving objects. Thus, their surface activities can hardly be visually-guided.

Taken together, the neuroanatomical findings suggest conservation of basic visual capabilities, casting doubt on the long-lasting notion that the African mole-rats are blind [20], [21]. In addition, they show that the visual system of the African mole-rats is neither suited for above-ground spatial orientation nor adapted for low-light vision. Therefore, it has been suggested that the main function of the residual, low acuity vision is to localize breaches in the burrow systems that let in light [7], [8], [12], [22].

In contrast to this wealth of information on organization of bathyergid visual system, almost nothing is known about their visual capacities. Recently, a single study has reported a light/dark discrimination ability in the Zambian mole-rats, *Fukomys anselli/kafuensis* [22]. Likewise, no experimental studies testing the adaptive significance of vision in the context of bathyergid anti-predatory behaviour are available. In this study, we investigated the visual capacities and the role of vision in two other Afrotropical mole-rats, the social giant mole-rat *Fukomys mechowii* and the solitary silvery mole-rat *Heliophobius argenteocinereus*. Firstly, we tested whether these species are able to perceive full-spectrum light and monochromatic lights of different wavelengths. Secondly, we examined their possible colour discrimination abilities. Finally, we tested the hypothesis that vision plays a role in bathyergid anti-predatory behaviour and tunnel maintenance by assessing whether light penetrating into an artificial tunnel system provokes plugging of the illuminated tunnel.

## Results

### White light avoidance

**Figure 1:**
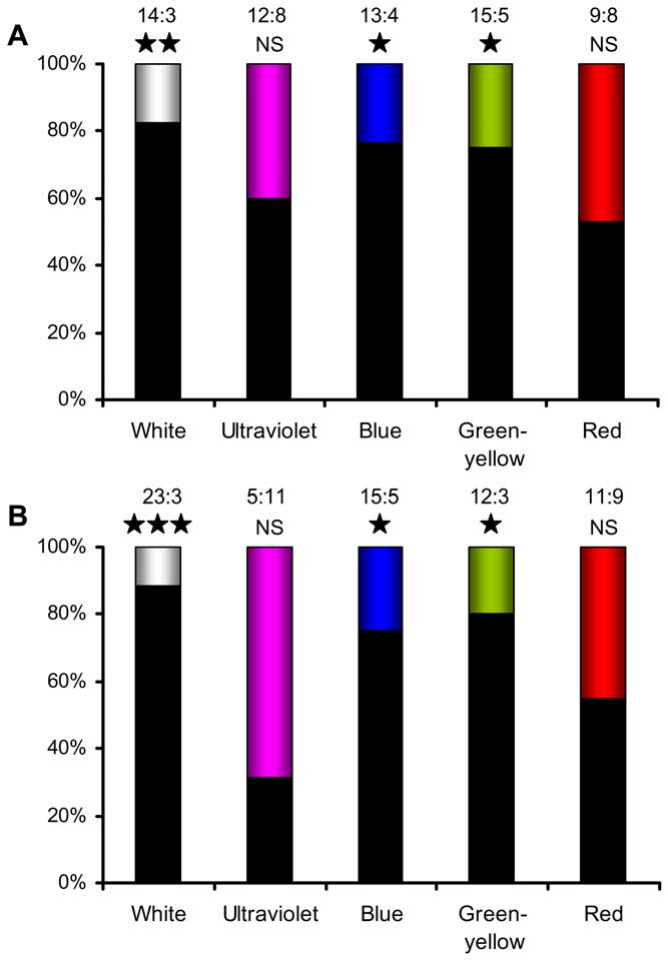
Light avoidance in the giant mole-rat *Fukomys mechowii* (a) and the silvery mole-rat *Heliophobius argenteocinereus* (b). Black bars represent the percentage of choices towards the dark and coloured bars towards the illuminated arm. The absolute number of choices made (dark: illuminated) and the statistical significance level of the response to the corresponding light are shown above each bar (***, P<0.001; **, P<0.01; *,P<0.05; NS, non significant).

The mole-rats showed clear heliophobic behaviour. Both species avoided the illuminated box and nested significantly more often in the dark box (*F*. *mechowii*: χ^2^ = 7.1, P<0.01, *N* = 17; *H*. *argenteocinereus*: χ^2^ = 15.4, P<10^−4^, *N* = 26; Fig. 1, left bars).

### Photoavoidance response to blue and green light

Both species preferred nesting in the dark box and avoided box illuminated by blue (*F*. *mechowii*: χ^2^ = 4.8, P = 0.029, *N* = 17; *H*. *argenteocinereus*: χ^2^ = 5.0, P = 0.025, *N* = 20) and green-yellow light (*F*. *mechowii*: χ^2^ = 5.0, P = 0.025, N = 20; *H*. *argenteocinereus*: χ^2^ = 5.4, P = 0.020, N = 15) (Fig. 1, third and fourth left bars). By contrast, animals showed a random choice between the dark box and the box illuminated by red light (*F*. *mechowii*: χ^2^ = 0.06, P = 0.81, *N* = 17; *H*. *argenteocinereus*: χ^2^ = 0.2, P = 0.65, *N* = 20; Fig. 1, right bars).

### No evidence for UV sensitivity

**Figure 2:**
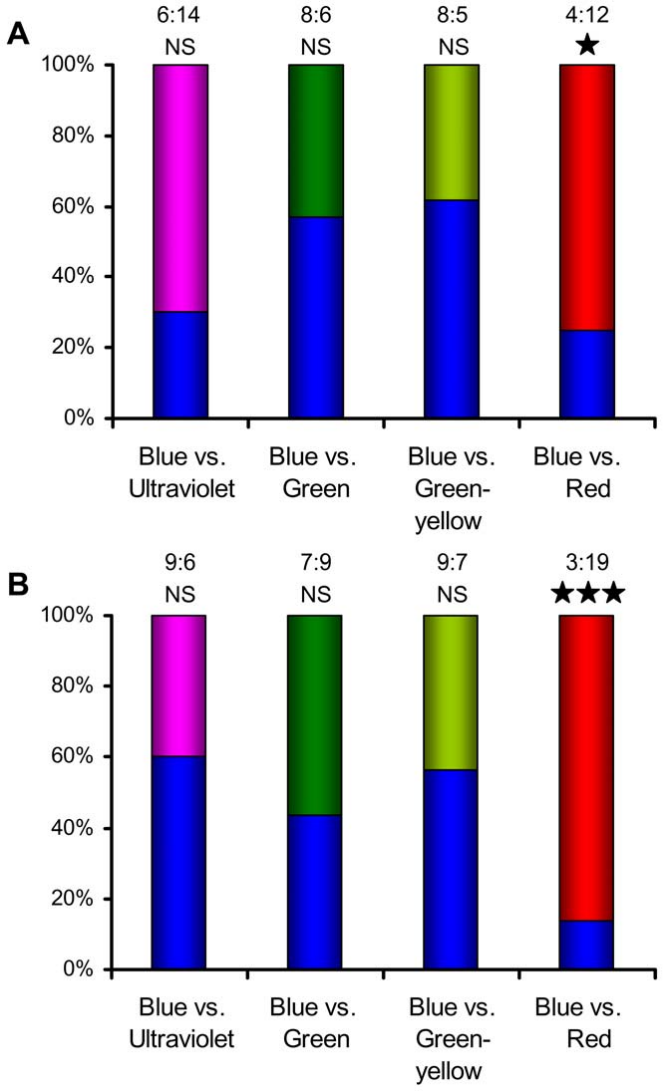
Results of colour preference test in the giant mole-rat *Fukomys mechowii* (a) and the silvery mole-rat *Heliophobius argenteocinereus* (b). Coloured bars represent the percentage of choices towards the respective colours. The absolute number of choices made (blue: other colour) and the statistical significance level of the response to the corresponding choice between two colours are shown above each bar (***, P<0.001; *, P<0.05; NS, non significant).

Both species showed a random choice between the dark box and the box illuminated by UVA light (*F*. *mechowii*: χ^2^ = 0.8, P = 0.37, *N* = 20; *H*. *argenteocinereus*: χ^2^ = 2.25, P = 0.13, *N* = 16; Fig. 1, second left bars). Surprisingly, the animals exhibited a random choice between the blue and UVA illuminated boxes (*F*. *mechowii*: χ^2^ = 3.2, P = 0.07, *N* = 20; *H*. *argenteocinereus*: χ^2^ = 0.6, P = 0.44, *N* = 15; (Fig. 2, left bars), although trend towards preference of the UVA illuminated box was seen in *F. mechowii*.

### No evidence for colour discrimination

In trials where boxes were illuminated by two different monochromatic lights, the mole-rats significantly preferred the box illuminated by red light to the box illuminated by blue light (*F*. *mechowii*: χ^2^ = 4.0, P = 0.045, *N* = 16; *H*. *argenteocinereus*: χ^2^ = 11.63, P<0.001, *N* = 22; Fig. 2, right bars), but exhibited a random choice between blue and green light (*F*. *mechowii*: χ^2^ = 0.28, P = 0.59, *N* = 14; *H*. *argenteocinereus*: χ^2^ = 0.25, P = 0.62, *N* = 16) and between blue and green-yellow light (*F*. *mechowii*: χ^2^ = 0.69, P = 0.41, *N* = 13; *H*. *argenteocinereus*: χ^2^ = 0.25, P = 0.62, *N* = 16) (Fig. 2, middle two bars).

### Light induced burrow plugging behaviour

**Figure 3:**
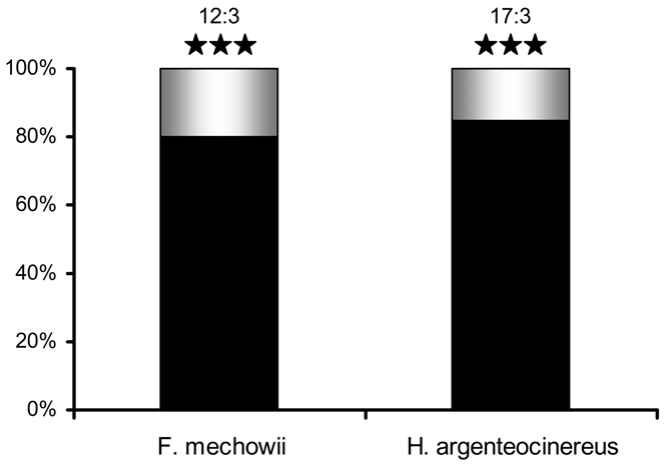
Burrow plugging behaviour. Black and white bars represent the percentage of trials in which experimental animals did and did not plug the illuminated burrow, respectively. The absolute number of trials and the statistical significance level of the response are shown above each bar (***, P<0.001).

*Fukomys mechowii* and *Heliophobius argenteocinereus* blocked the illuminated tunnel with peat in 80% and 85% of trials, respectively (Fig. 3). The former species did on average 2.0, the latter species 1.75 plugs per trial. Since the maze consisted of seven blind tunnels, the probabilities that the illuminated tunnel will be blocked by chance are 2.0/7 and 1.75/7, respectively. Consequently, if mole-rats plug blind tunnels accidentally, the blocking of the illuminated tunnel is expected in ∼29% and 25% of trials, respectively. Both mole-rat species thus plugged the illuminated tunnel significantly more often than expected by chance (*F. mechowii*: χ^2^ = 18.4, P<10^−4^, N = 15; *H. argenteocinereus*: χ^2^ = 38.4, P<10^−9^, N = 20). In addition, plugs located within the illuminated tunnel were longer and more tightly packed with peat than those located within the dark tunnels (data not shown).

## Discussion

The preferential nesting assays performed in this study show that two bathyergid species, the social giant mole-rat *Fukomys mechowii* and the solitary silvery mole-rat *Heliophobius argenteocinereus*, exhibit a clear photoavoidance response to white, blue and green-yellow light, but no significant reaction to ultraviolet or red light. The mole-rats thus retain dark/light discrimination capabilities and a capacity to perceive short to medium-wavelength light. These findings further imply that the mole-rat S opsin has its absorption maximum in the blue rather than the UV part of the spectrum. The assay did not yield conclusive evidence regarding colour discrimination. Finally, the blocking of illuminated tunnels reported here suggests that light serves as a cue signalling the damage of the tunnel system, and therefore points to an important role of vision in bathyergid anti-predatory behaviour and tunnel maintenance.

### Dark/light discrimination

Among strictly subterranean mammals, light avoidance behaviour has been reported in five species of African mole-rats [present study, 12, 22], the blind mole-rat *Spalax ehrenbergi* [23], and two species of insectivore talpid moles – *Talpa europea* and *T. occidentalis* – [24]–[27]. It is notable that *S*. *ehrenbergi* and *T. occidentalis* posses completely subcutaneous eyes with a degenerated optical apparatus [28]–[30] and thus represent the extreme cases of eye regression. Consequently, the capacity to distinguish between light and darkness seems to be a common trait amongst subterranean mammals.

### Rod and cone opsins, spectral sensitivity and colour discrimination

Bathyergids have a unique photoreceptor mosaic consisting of rods (∼90% of photoreceptors), dual pigment cones coexpressing S and L opsins (∼7%), pure S cones (∼2%) and pure L cones (∼1%) [11]. Hence, the mole-rat retina is equipped for both rod scotopic (low light) and cone photopic (daylight) vision. The two spectrally different cone types may subserve dichromatic colour vision, provided that the appropriate post-receptor retinal and cortical circuits for colour processing are also preserved. However, the expression level of the S opsin by far exceeds that of the barely detectable L opsin [11]. The S opsin dominance supports a greater short-wavelength sensitivity than mid-wavelength sensitivity. Nevertheless, the avoidance of both blue and green-yellow light, and a random choice between blue and green light and between blue and green-yellow light demonstrated here, clearly show that that mole-rats are able to see blue as well as green and green-yellow light. These findings raise the question as to whether the green/green-yellow light sensing is L cone- or rod-mediated.

The green light (λmax  = 507 nm) irradiance of 5 µmol photons s^−1^ m^−2^ corresponds to a luminance of ∼640 scotopic candelas m^−2^. For a human eye, this luminance is at least 1 log unit higher than that needed for rod saturation [31]. A green light of this intensity produces ∼3.72×10^5^ and ∼1.16×10^4^ photoisomerizations per rod per second (Rh* rod^−1^ s^−1^) in the mouse with a fully dilated and fully constricted pupil, respectively [32]. Because the mouse pupil is fully constricted under these light levels [33], the latter estimate is more realistic. The threshold for cone activation is ∼30 Rh* rod^−1^ s^−1^ [34]. In the rat, rod saturation occurs at ∼4×10^3^ Rh* rod^−1^ s^−1^ [35]. In a transgenic mouse whose retina lacks cones, the ganglion cell response greatly attenuates at the intensity of ∼10^4^ Rh* rod^−1^ s^−1^ and disappears at intensities above 10^5^ Rh* rod^−^1 s^−1^ [36]. One may speculate that bathyergid mole-rats are even more sensitive to rod saturation caused by bright light than surface-dwelling rodents. Their eyes are smaller than those of mouse, so that an equivalent amount of light passing the murine and mole-rat pupils is spread over a ∼3–5-fold smaller area on the mole-rat retina ([37]; Němec et al., unpublished data). Moreover, the rods of mole-rats have substantially larger inner and outer segment diameters when compared to rat or mouse [11]. Consequently, more photons are funnelled to an individual rod in the mole-rats. At the same time, bathyergid rods have shorter outer segments with less densely packed discs (the opsin-containing structures) than sighted rodents [10], suggesting that the total amount of rhodopsin per rod may actually be smaller in bathyergids. As a result, ambient light of any given intensity would bleach a higher fraction of rhodopsin in the bathyergid mole-rats. Assuming that the sizes of fully constricted pupils and photon capture efficiencies of rods are comparable in mouse and bathyergids, one can roughly estimate (using the formulas published by Lyubarsky et al. [32]) that the green light used in this study produces ∼1.7×10^5^ Rh* rod^−1^ s^−1^ and ∼1.0×10^5^ Rh* rod^−1^ s^−1^ in *H. argenteocinereus* and *F. mechowii*, respectively. Taken together, the light intensity used in our experiments is clearly in the photopic range, where cone signals dominate and rods contribute little, if anything, to the ganglion cell response. It is therefore very likely that L cones and/or dual-pigment cones mediated the perception of green/green-yellow light in our experiments. Thus, the function of both S and L cones seem to be conserved in the African mole-rats.

Animals chose randomly between the dark box and the box illuminated by red light, and preferred red light significantly when had to choose between the boxes illuminated by red and blue light, implying that they cannot see the red light. This result, however, is not surprising. The rodent L cone pigment is commonly green- or yellow-sensitive with λ_max_ somewhere in the range of about 495 to 535 nm (e.g., [38], [39]). Indeed, far red light was intentionally used as a control condition for assay validation. The fact that mole-rats did not avoid UVA light is less trivial. The rodent S cone pigment is either UV- or violet/blue-sensitive, depending on the species (e. g., [40], [41]). The task, where animals had to make a choice between the dark box and the box illuminated by monochromatic light, brought clear results: the animals avoided blue light but not UVA light. This finding strongly suggests that the bathyergid S cone pigment is violet/blue-sensitive. The other task, where animals had to choose between the boxes illuminated by blue and UVA light, were less conclusive. While an obvious (albeit not significant) bias towards the preference of UVA light was observed in *F. mechowii*, *H*. *argenteocinereus* exhibited a random choice between blue and UVA light. The interpretation of the latter puzzling result is currently unclear. One possibility is that the S opsin of *H*. *argenteocinereus* has its λ_max_ in violet and its absorbance spectrum spreads well below 400 nm. But whatever the reason, the data obtained in this study altogether suggest that the bathyergid S opsin is violet/blue-sensitive.

The preferential nesting experiments provided no evidence for colour discrimination. As noted above, both species chose randomly between nest boxes illuminated by blue and green light and between boxes illuminated by blue and green-yellow light. However, this assay is entirely based on the spontaneous motivation to avoid light. While the evidence for heliophobic (or scotophilic) behaviour is compelling ([22], present study), it remains unclear whether mole-rats would spontaneously prefer either monochromatic light if they were capable to discriminate between them. Hence, the negative results of the performed behavioural tests are inconclusive. It has to be noted in this context, that the very fact that the majority of bathyergid cones coexpress S and L opsins may compromise but does not necessarily preclude colour vision. For instance, mice are able to discriminate colours [42] despite having a substantial population of dual pigment cones [43]. A relatively small number of pure S-cones and L-cones may be sufficient to support dichromatic colour vision. Actually, the proportion of 3% for the spectrally distinct cones outnumbers the proportions in many nocturnal species having between 0.5 and 3% cones among their photoreceptors (for overviews, see [40], [44]). Conditioning experiments will be needed to assess the capacity for colour vision in the bathyergid mole-rats.

### Possible role of vision in the ecology of mole-rats

The African mole-rats have adopted a subterranean mode of life during the early Miocene, if not earlier [9]. Why do the African mole-rats retain basic visual capabilities even after millions of years of underground existence? Despite a remarkable progress in the understanding of the constrains imposed on bathyergid visual capacities by their minute eyes and a reduced visual system (for review, see [7], [8]), the biological significance of vision in the natural environment of the bathyergid mole-rats is uncertain. Hypothetically, vision may contribute to the fitness of bathyergids in three ways.

First, it is well known that some subterranean rodents, including the African mole-rats, use light dark cycle as Zeitgeber to which circadian activity is entrained under laboratory conditions [45]–[51]. However, it is unclear as to whether such light entrainment occurs in the nature. Since light does not penetrate into sealed underground burrows effectively enough to provide a perceivable cue, it is generally expected that strictly subterranean rodents come commonly into contact with light only during forming mounds. However, at least in some species, this activity is rather irregular. For instance, *H. argenteocinereus* cease mound building during dry season [52] and some individuals produce mounds occasionally with no appearance of new mounds for several weeks/months [53]. In this case, the synchronization of circadian activity with the ambient photoperiod would be probably difficult. Therefore some other environmental factors may act as Zeitgeber. Indeed, the results of a recent radiotracking study suggest that soil temperature could be a better predictor of circadian activity rhythms [54]. The fact that c-Fos expression in the suprachiasmatic nucleus is not gated according to the phase of the circadian clock in some social bathyergid species [55], [56] also indicates a reduced photic sensitivity of the mole-rat circadian system.

Second, vision might be useful for guidance and/or timing of rare surface activities. Apart from non-recurring events such as natal dispersal, emigration after depleting of food resources, flooding etc., more regular surface activities such as searching for mates during mating season have been recently reported [57]. In *H. argenteocinereus*, paternity analyses demonstrated that burrow systems of mating pairs were several hundred meters away from each other in some cases. The absence of any belowground connection between mate tunnel systems, and a female biased sex ratio imply that aboveground seeking for a mate is a part of the mating strategy at least in one sex [57]. However, as mentioned above (see Introduction), the extremely low visual acuity and severe regression of the visual domains involved in the coordination of visuomotor reflexes render bathyergid above-ground visually guided navigation and predator avoidance ineffective if not impossible.

Nevertheless, vision may be used to optimize the timing of above-ground excursions. Light intensity as well as illuminant spectra change depending on the time of the day. Hence, different photoreceptors are preferentially stimulated at noon, in the twilight or at night. For example, twilight has a higher level of short-wave components than daylight or moonlight [58]. Thus, S cone dominance may possibly optimize the quantal capture and hence cone primary vision at twilight. However, the underground life is associated with markedly different patterns of cone opsin expression and spectral sensitivity among phylogenetically distant taxa [11], [59]–[62]. More detailed information about species-specific frequencies of light exposure and light related behaviour patterns will be required to asses whether S cone dominance confers any selective advantage to the bathyergid mole-rats. Given that cones switch expression from the S to L opsin in some species during early postnatal development (for review, see [63]), it cannot be currently excluded that the S cone dominance is a mere consequence of arrested cone development.

Third and finally, the adaptive significance of vision may be related to an anti-predatory behaviour [7], [8], [12], [22]. The incidence of light may signal that a burrow is damaged by predators (or incidentally by the activity of large herbivores, rains, etc.) and warn the belowground dweller not to approach the opening too closely. Indeed, the very cautious behaviour of *H. argenteocinereus* approaching damaged burrows in the field suggests that mole-rats are aware of burrow violation well before they reach the damaged place (cf. [64]). Many subterranean rodents, including the African mole-rats, react to damage of their tunnels by blocking the broken part with soil. But does light really act as a cue eliciting this behaviour? Beside light, noise from outside, a change in humidity and/or temperature or increased ventilation in the vicinity of the damage may indicate breaches in the burrow. While all these cues may act in synergy in the nature, here we show that the presence of light per se induces very effectively plugging behaviour under laboratory conditions. Accordingly, light was reported to be the primary cue entraining plugging behaviour also in the pocket gophers, phylogenetically unrelated subterranean rodents that posses large eyes [65]. The ease of demonstrating light induced tunnel blocking in a laboratory experiment suggests firm coupling between light stimuli and the plugging behaviour. We therefore conclude that vision does play an important role in bathyergid anti-predatory behaviour and tunnel maintenance.

## Materials and Methods

### Animals

The silvery mole-rat (*Heliophobius argenteocinereus*, Peters 1846) inhabits southern Kenya, Tanzania, Malawi, southeast D. R. Congo, eastern Zambia, and northern Mozambique; the giant mole-rat (*Fukomys mechowii*, Peters 1881) inhabits northern Zambia, south D. R. Congo, and Angola. Both model species feature very similar ecologies but differ starkly in their life histories: the silvery mole-rat is solitary while the giant mole-rat is a social cooperative breeder. Their biology has been reviewed recently [66], [67].

A total of 26 silvery mole-rats and 44 giant mole-rats were used in this study. The silvery mole-rats were wild caught in Malawi in Mpalanganga estate, Zomba (15° 27′S, 35° 15′E), Zomba plateau (15° 20′S, 35° 16′E), and Mulanje - Chipoka (16° 02′S, 35° 30′E) in 2000 and 2005. Some of the giant-mole rats were caught in 1999 in Ndola in Zambia, but the rest was born in captivity. The animals were reared and/or kept in an animal room with moderate temperature (25±1°C) and a 12L/12D light regime at the University of South Bohemia. The silvery mole-rats were housed individually in plexiglass mazes, the families of the giant mole-rats in terrariums. The mole-rats were fed with carrots, potatoes, lettuce, apples, and rodent pellets. Animals at least one year old were tested. Each mole-rat was tested only once in each test condition. The social giant mole-rats were tested in pairs (or threesomes) to avoid stress from isolation. In this species, the availability of experimental animals was periodically influenced by breeding. In the silvery mole-rat, the sample sizes gradually decreased in the course of testing due to the mortality of the experimental animals (this is to be noted in this context that it took three years to perform all experiments).

#### Ethic statement

All experiments were approved by Institutional Animal Care and Use Committee at University of South Bohemia and Ministry of Education, Youth and Sports (n. 12924/2007-30).

### Experiment 1: preferential nesting assay

**Figure 4:**
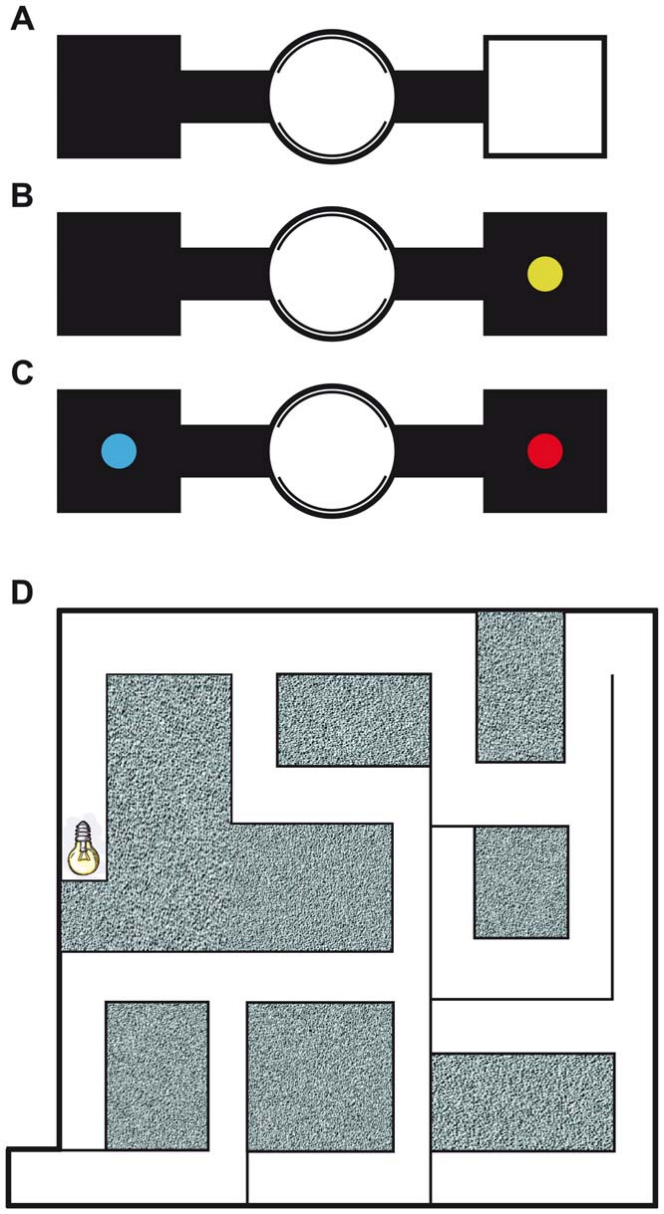
The experimental paradigms used in this study. (a–c) Different layouts of a binary-choice apparatus for testing nest building preference. (d) Diagram of an artificial burrow system used to evaluate burrow plugging behavior; the bulb icon marks the illuminated tunnel. A detailed description of the mazes and experimental protocols are given in the text.

The abilities to discriminate between full-spectrum (“white”) light and darkness, to perceive monochromatic lights of various wavelengths and to discriminate colours were tested using a preferential nesting assay [39], [42]. Mole-rats were allowed to choose between a dark box and a box illuminated by full-spectrum light (Fig. 4a), between a dark box and a box illuminated by monochromatic light (Fig. 4b), and between two boxes illuminated by two different monochromatic lights (Fig. 4c), respectively.

A binary-choice apparatus (Fig. 4a-c) was made of plastic and consisted of a cylindrical centre (diameter 22 cm, height 35 cm), an inner cylinder providing opening and closing of the tunnels (diameter 20 cm, height 30 cm), two opposite tunnels (15×8×8 cm) with two terminal boxes (20×20×20 cm). The boxes could be covered with an opaque plastic lid, a translucent Plexiglass lid or an opaque plastic lid with a central opening (5 cm in diameter) for insertion of spectral filters. In every test, the position of lids was swapped after each trial. To begin each experiment, animals were placed into the closed cylindrical centre. Three pieces of carrot, pellets and nesting material (8 strips 25×5 cm of filter paper) were provided. Then the inner cylinder was rotated so that animals could enter and explore the maze. A result was recorded when all nesting material was found in one box (boxes were checked after 60 and 90 minutes). Between trials, the whole apparatus was thoroughly cleaned with ethanol.

**Figure 5:**
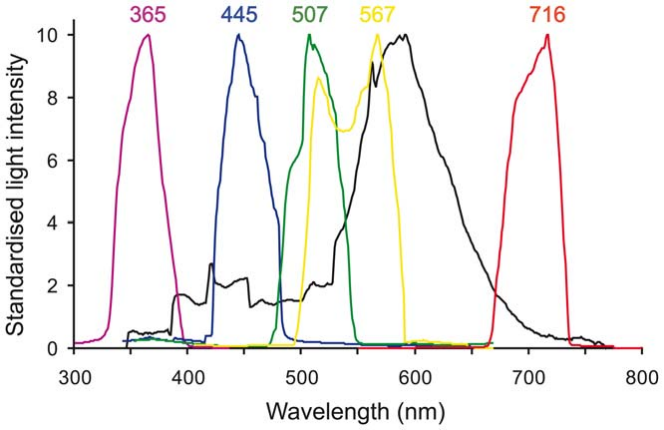
Spectra of the full-spectrum and monochromatic lights used in the experiments. The black line indicates the spectrum of the full-spectrum light produced by fluorescent tubes; coloured lines indicate the spectra of the monochromatic lights (note that the colour-code is symbolic, i.e., the colours do not exactly match to the spectra). The peak wavelengths are given for monochromatic lights. For clarity, all spectra are standardised by taking the maximum value within the measured wavelength interval as 10.

Two fluorescent tubes (OSRAM L 58 W/31-830) were used to produce full-spectrum light (400–750 nm) (Fig. 5). The apparatus was illuminated from a distance of 130 cm. The light intensity at the bottom of the illuminated sites was 10 µmol photons s^−1^ m^−2^, as measured by a data logger (Minikon QT, EMS, Czech Republic). To produce monochromatic lights of various wavelengths, 40 W incandescent light bulbs were used in combination with colour filters (diameter 50 mm, thickness 5 mm, Chroma Technology Corp., Rockingham, USA) representing the following parts of the visible spectrum: blue (420–490 nm), green (470–550 nm), green-yellow (495–590 nm) and red (665–735 nm) (Fig. 5). A glass Petri dish filled with cold water was placed above each lid to absorb any heat radiation from the bulbs. The position of the bulb was adjusted according to the type of filter used in order to achieve the light intensity of app. 5 µmol photons s^−1^ m^−2^ at the bottom of each nest box. To produce UVA light (350–400 nm), a UV lamp (UVP, Inc., Upland, USA) was used in combination with a UV-pass filter (Jos. Schneider Optische Werke GmbH, Bad Kreuznach, Germany). The light intensity was set to 1 µmol photons s^−1^ m^−2^ to minimize the possible detrimental effect of UV light on the experimental animals. The intensity of the UV light was measured using a PD300-1W/1Z02411 photodiode sensor (Ophir Optronics Ltd., Israel). Light spectra were determined using Avaspec 2048 Fiber Optic Spectrometer (Avantes BV, Eerbeek, The Netherlands). The temperature in each box was measured after each trial using a probe thermometer (TESTO 425). No difference in temperature was found between boxes. The cylindrical centre of the maze was illuminated by full-spectrum light with an intensity of 10 µmol photons s^−1^ m^−2^ in all experiments.

### Experiment 2: burrow plugging behaviour

In this experiment, the reaction of mole-rats to light penetrating into a maze simulating a natural burrow system (Fig. 4d) was tested. If the incidence of light serves as a signal of a damaged burrow and mole-rats are capable of detecting this alert cue visually, they should fill the illuminated part of the maze with substrate.

The maze was made of transparent plexiglass, measured 110×100×10 cm and consisted of seven blind tunnels. The whole maze was tightly closed with a transparent lid. Before the experiment, this lid was covered with black paper except for the end of one blind tunnel that remained transparent. During experiments, this end was illuminated by a 40 W incandescent light bulb from a distance of 130 cm. The animals were introduced into the maze with a thin layer (∼1 cm) of horticultural peat and food provided. Subsequently, the maze was covered and the transparent end of the tunnel exposed to light. Each animal (pairs or threesomes in case of the giant mole-rats) spent two hours in the maze. Afterwards, we recorded whether the experimental animals plugged the illuminated tunnel.

### Data analysis

In all experiments, we used chi-square tests to analyze the data for a preferential choice. In the Experiment 1, a random choice was asserted by the null hypothesis, i.e., the expected (theoretical) frequency was 1∶1. In the Experiment 2, the null hypothesis asserted that the illuminated tunnel in the experimental maze was plugged by chance. The probability the probability of the illuminated tunnel being plugged was calculated as the ratio of the mean number of plugs per trial (Plug_mean_) to the total number of the blind tunnels in the maze (there is a one in seven chance that the illuminated tunnel will be plugged). Thus the expected frequency of the illuminated tunnel plugging was computed as follows: N× Plug_mean_/7. Consequently, the expected frequency of the presence: the absence of the plug in the illuminated tunnel was N× Plug_mean_/7: N× (1 – Plug_mean_/7).

A 95% confidence level (P<0.05) was used to judge statistical significance. Analyses were performed using STATISTICA for Windows (StatSoft, Inc., Tulsa, OK, USA).
